# Angina Pectoris and Pseudoangina

**Published:** 1905-06-17

**Authors:** 


### ANGINA PECTORIS AND PSEUDO-ANGINA.

In an address on this subject, Sir Wm. Broadbent[1] says that when attacks of paroxysmal pain in the cardiac region come on chiefly during repose—for example, when the patient is reading or writing—however severe they may be, and however closely they may appear to conform to true angina, the presumption is that they are spurious unless they have been led up to by the frequent occurrence of pain induced by exertion. This presumption is increased if there are also tumultuous action of the heart and irregularity or intermission of the pulse, of which the patient is conscious, particularly if the pain lasts for some time. The same conclusion is indicated by cardiac pain occurring during the night, if there is no history of the patient having also been brought up short when walking by pain in the chest. Further, if nocturnal attacks recur, and more particularly if they observe a sort of punctuality in their recurrence, it may be concluded that they are of gastric origin. In all these circumstances, however, the existence of valvular disease of the heart or of considerable cardiac dilatation will demand caution in dismissing the attacks as free from serious significance. The condition which most closely simulates true angina is paroxysmal cardiac pain associated with dilatation of the stomach, especially when at the same time there exists high arterial tension. In illustration of this simulation of true angina Sir William quotes the case of a retired officer æt. 70 years, who on several occasions was pulled up when walking by pain in the chest which extended down each upper limb. Other features of the attack were breathlessness and palpitation, and the patient often had to lie flat on the floor. This last fact was decidedly against a diagnosis of true angina in which, also, a complaint of palpitation is very unusual. The patient had similar attacks at 3 a.m., and on examination was found to have a much dilated stomach and normal heart sounds. Under dietetic treatment, with medicines to reduce arterial tension and to relieve distension of the stomach, he made a prompt recovery.

As opposed to the above facts the diagnostic distinction of true angina is that the attacks of pain are brought on by exertion. Its clinical associations are aortic valvular disease, aortitis, dilatation of the aorta (general or aneurysmal), atheroma of the aorta, and fatty degeneration of the heart. It is when the coronary arteries are implicated that the attacks of angina become established. In a large proportion of cases arterio-sclerosis is present in a marked degree. This is seen in firm, tortuous radial and brachial arteries, the vessel remaining full between the beats. Dilatation of the aorta gives a loud, low-pitched, sometimes ringing, aortic second sound. But attacks of angina may occur before any of these physical changes in the arterial system can be appreciated, in consequence of early interference with the coronary circulation. These are on the whole unfavourable cases, as are also those attended with fatty degeneration of the heart. The treatment is that of high arterial tension. This is the result of resistance in the peripheral circulation excited by the presence of toxins. Hence the diet must be simplified, the supply of meat reduced, and all meat extracts forbidden. Even a strict milk diet may be necessary for a time. The free use of water, aided, perhaps, by the potassium or lithium salts, is useful as an aid to elimination, and good hygienic conditions and moderate daily exercise should be encouraged. Mild mercurial aperients and the iodides are more definite agents in the reduction of arterial tension, and amyl nitrite and nitro-glycerine are invaluable in the actual paroxysmal seizures. Erythrol tetranitrate has a similar but more sustained effect in relieving vascular spasm. The treatment of any existing dyspepsia, and more particularly the prevention of flatulent distension of the stomach, are of the first importance. This latter condition in cases of angina or of any serious heart disease may be the immediate cause even of a fatal issue. In any case it may contribute to bring on attacks. The same is true of a hearty meal. Hence the importance of forbidding exercise shortly after food.

[1] Lancet, May 27, 1905.

